# Supplementary material for: Insights from aquaporin structures into drug-resistant sleeping sickness
Source: eLife. 2026 Jan 14;14:RP107460. doi: 10.7554/eLife.107460 (PMC12803511; doi:10.7554/eLife.107460)
Supplement: Supplementary file 1. [file elife-107460-supp1.docx]

**Supplementary file 1 | Details of molecular dynamics simulations on TbAQP2.**

| **System** | **Box size** | **No. atoms** | **Setup** | **Simulation length** |
| --- | --- | --- | --- | --- |
| AQP2 WT tetramer | 10x10x10 nm | ca. 95,000 | Pentamidine | 5 x ca. 300 ns |
| AQP2 WT tetramer | 10x10x10 nm | ca. 95,000 | Apo | 5 x ca. 300 ns |
| AQP2 WT monomer | 6.5x6.5x10 nm | ca. 45,000 | Pentamidine | 5 x 800 ns |
| AQP2 WT monomer | 6.5x6.5x10 nm | ca. 45,000 | Pentamidine and +ve electric field | 3 x ca. 600 ns |
| AQP2 WT monomer | 6.5x6.5x10 nm | ca. 45,000 | Pentamidine and -ve electric field | 3 x ca. 1400 ns |
| AQP2 WT monomer | 6.5x6.5x10 nm | ca. 45,000 | Pentamidine steered MD | 1 x 5 ns pull |
| AQP2 WT monomer | 6.5x6.5x10 nm | ca. 45,000 | Pentamidine PMF | 167 x 40 ns windows |
| AQP2 WT monomer | 6.5x6.5x10 nm | ca. 45,000 | Apo | 5 x 800 ns |
| AQP2_L258Y_L264R_ monomer | 6.5x6.5x10 nm | ca. 45,000 | Pentamidine | 5 x 800 ns |
| AQP2_L258Y_L264R_ monomer | 6.5x6.5x10 nm | ca. 45,000 | Pentamidine steered MD | 1 x 5 ns pull |
| AQP2_L258Y_L264R_ monomer | 6.5x6.5x10 nm | ca. 45,000 | Pentamidine PMF | 167 x 40 ns windows |
| AQP2_L258Y_L264R_ monomer | 6.5x6.5x10 nm | ca. 45,000 | Apo | 5 x 800 ns |
| AQP2_I110W_ monomer | 6.5x6.5x10 nm | ca. 45,000 | Pentamidine | 5 x 800 ns |
| AQP2_I110W_ monomer | 6.5x6.5x10 nm | ca. 45,000 | Pentamidine steered MD | 1 x 5 ns pull |
| AQP2_I110W_ monomer | 6.5x6.5x10 nm | ca. 45,000 | Pentamidine PMF | 167 x 40 ns windows |
| AQP2_I110W_ monomer | 6.5x6.5x10 nm | ca. 45,000 | Apo | 5 x 800 ns |
